# Revisiting the Tsunami: Health Consequences of Flooding

**DOI:** 10.1371/journal.pmed.0020184

**Published:** 2005-06-28

**Authors:** Oliver Morgan, Mike Ahern, Sandy Cairncross

## Abstract

Morgan and colleagues critically review the evidence on the health consequences of flooding disasters, and consider what interventions are appropriate.

**Figure:**
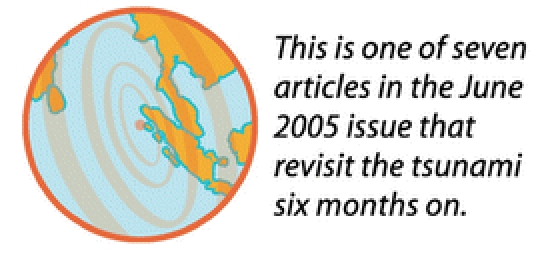


The South Asian tsunami on 26 December 2004 was one of the largest flooding disasters in recent history (Figure 1), causing about 280,000 fatalities in eight countries stretching from Asia to Africa [1]. Shortly after the disaster, the World Health Organization warned that disease could claim as many lives as the tsunami itself [2].

**Fig 1:**
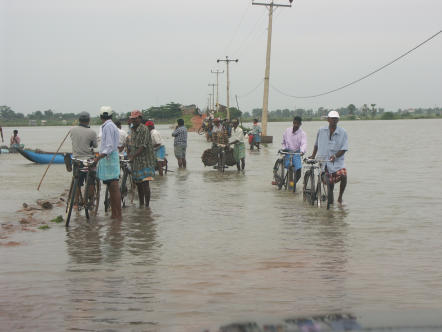
Flooding on the East Coast of Sri Lanka after the Tsunami (Photo: Copyright, Aur lie Gr maud/MSF)

Early-warning communicable disease surveillance systems were established in the affected areas. However, in the following weeks, no large disease outbreaks were reported. In this article, we review the evidence for the health consequences of flooding disasters and consider what interventions are appropriate.

## The Evidence on Flooding and Health

A recent systematic review of published literature found limited epidemiological evidence about the health effects from flooding disasters [3]. Notably, there were fewer studies from developing countries, where the disease burden is likely to be higher.

### Diseases transmitted by the faecal–oral route.

**Fig 2:**
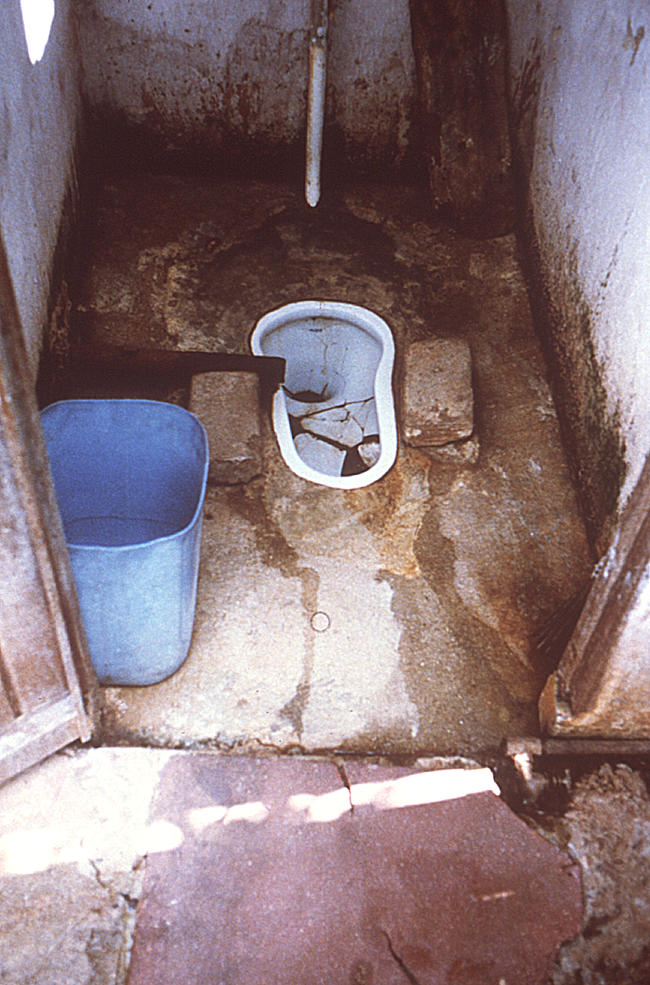
Conditions inside the Water Closet of the Index Case Residence during a Typhoid Outbreak in Cite Roche Bois, Mauritius Salmonella typhi–contaminated sewage was aspirated into the main water distribution system after Hurricane Claudette's flooding, evidenced by high water marks on the walls. Note the leaking water pipe on the rear wall, and general unsanitary conditions. (Photo: CDC)

The review found that diseases transmitted by the faecal–oral route were the main flood-related health impact. Such diseases include nonspecific diarrhoea, cholera, dysentery, and typhoid (Figure 2) [4]. For example, diarrhoea increased by a factor of two to four after flooding in Mozambique during 2000 [5]. Although increased incidence of diarrhoeal disease in affected populations is not usually associated with increased mortality, there have been some exceptions. Flooding in West Bengal in 1998 was followed by an outbreak of diarrhoea, suspected to be cholera, which resulted in 16,590 cases and 276 deaths (case–fatality ratio 1.7%) [6].

### Mosquito-borne diseases and other infections.

Flooding may also create a large number of breeding sites for mosquito-borne diseases such as malaria, and there have been numerous reports of increased incidence in previously endemic countries throughout Africa, Asia, and Latin America. This increase can be particularly important when populations are displaced. After the Mozambique floods of 2000, the number of malaria cases within the displaced population increased by a factor of 1.5 to two times previous levels [5].

Outbreaks of leptospirosis, a zoonotic bacterial disease associated predominantly with rats, have occurred when mud and water are contaminated by the urine of infected rodents. Flooding in Guyana in February 2005 led to more than 40 cases of leptospirosis. Other nonspecific infections such as conjunctivitis and ear, nose, and throat infections also increase [7].

After natural disasters, the media, health professionals, and relief workers often say that dead bodies of victims can cause epidemics of diseases such as cholera [8]. The fear caused by these claims encourages communities, local authorities, and governments to rapidly dispose of victims without identification. This contributes to psychological distress among surviving relatives and creates legal problems where there are property, inheritance, or insurance claims. However, victims of natural disasters die from trauma, burns, or drowning and are unlikely to harbour pathogenic organisms such as cholera, which can cause epidemics [9]. For the public, the risk of infectious disease from dead bodies after natural disasters is negligible. Individuals handling cadavers may have a small risk of exposure to tuberculosis, blood-borne viruses (such as Hepatitis B or C and HIV), and gastrointestinal infections. However, the risk of infection can be greatly reduced by following basic hygiene precautions [9].

### Injuries.

The idea that flooding disasters cause large numbers of injuries is a common disaster myth that has regularly been proven to be wrong [10,11]. Even after violent flooding events such as the recent tsunami, the number of serious injuries is much lower than many medical emergency teams expect [11,12]. However, information about the number and type of injuries is often lacking, and improved data collection would improve our understanding of injury risk due to floods. Nevertheless, even in the absence of serious injury, wound infections of cuts and abrasions are common [7,12]. For example, in the Indonesian Province of Aceh, 106 cases of tetanus and 20 deaths were reported (case–fatality ratio 18.9%) after the tsunami at the end of 2004 (unpublished data).

### Mental health problems.

Mental health impacts, which include common mental disorders, post-traumatic stress syndrome, and suicide, have not been well documented [13]. Again, much of the existing evidence comes from Western countries [3], where coping mechanisms and cultural contexts are likely to be different than in many lower-income countries [14]. However, several studies have reported increased symptoms, such as anxiety, depression, and sleeplessness, among flood victims [3,15]. Behaviour change in children has also been observed, with Durkin et al. reporting increased bed-wetting and aggression [16], and other studies reporting post-traumatic stress disorder and dissatisfaction with life [3]. Only two studies have studied suicide among flood victims, and the evidence of an effect is unclear [3].

## Addressing the Health Consequences of Flooding

Appropriate and timely intervention can significantly reduce the risk of mortality and morbidity from infectious diseases after flooding disasters. In the short term, preventing the diseases spread via the faecal–oral route is the most important public health intervention. This relies on three measures: provision of clean water, suitable sanitation, and hygiene promotion (Figure 2) [17].

### Providing clean water.

Each person requires a minimum of 15 litres per day for drinking, cooking, and washing [17]. Re-establishing a basic supply of clean water in urban areas may be complicated by damage to water treatment works or distribution pipelines. Electricity distribution grids may also need repair to run water pumps. In rural areas, open wells and hand pumps are often easier to rehabilitate. However, lack of access to affected areas usually hampers response efforts; flooding makes roads impassable, and access to all affected areas can often take several days or weeks.

### Sanitation.

Sanitation is particularly important when affected populations seek shelter in communal settings such as schools. Additional latrines may have to be constructed in the short term. Household facilities such as pit latrines may be flooded or destroyed, often leaving returning communities without sanitation. Similar to urban water supply, urban sanitation systems are more complex and more costly to repair. Temporary low-tech solutions may be able to bridge the gap while longer-term repairs are made. It is much easier to promote such solutions among people who have owned toilets but lost them in a flood, than among communities unaccustomed to sanitation.

### Personal hygiene.

Personal hygiene is especially important when individuals have reduced access to clean water and sanitation or are living in crowded or temporary accommodation. Floodwaters often carry substantial faecal contamination, and people may need to be alerted to the need to clean all household possessions that they have touched. Hygiene promotion messages highlighting the importance of hand washing should be considered as necessary as provision of clean water [18]. Such activities should also be supported by provision of basic materials such as buckets and soap.

## Planning for the Future: Disaster Preparedness and Mitigation

It is often believed that casualties from natural disasters are unavoidable, but this belief is false [8]. There are many measures that can reduce morbidity and mortality following large and potentially catastrophic flooding events [19].

Early warning of impending floods and natural events such as hurricanes allows sufficient time for communities to be evacuated to safe areas. Although Florida experienced one of the worst hurricane seasons on record in 2004, the number of fatalities was lower than expected because of early warning and evacuation. Early warning also provides sufficient time to prepare when evacuation is not possible. Hurricane George in 1998 caused widespread damage and several fatalities in the Dominican Republic, where residents were not warned. In contrast, Cuba and Puerto Rico experienced relatively limited damage and loss of life, because preparations were made in the hours before the storm.

Disaster preparedness can also be developed for communities that are regularly exposed to flooding disasters. Cyclone shelters built by the Red Cross in Orissa, India, saved many thousands of lives in 1999 when two cyclones struck. In the region of the Americas, the Pan American Health Organization has spent many years promoting and integrating disaster preparedness into building health facilities to ensure that medical services needed to treat victims and maintain ongoing care for patients with chronic conditions will not be disrupted by disasters.

A recent global-scale review of health risks from flooding highlights that in flood-prone areas, disaster preparedness within the health system as a whole is particularly important [20]. In addition to infrastructure and early-warning systems, another key element in disaster preparedness is education and raising awareness about disaster risks and response plans. Had there been greater awareness about the risk of tsunamis, perhaps many lives could have been saved in the South Asian disaster in December 2004.

## Conclusion

Disasters such as the tsunami in South Asia underline the vulnerability of many communities in developing countries. Although the importance of clean water and sanitation get top billing during natural disasters, their absence during normal conditions carries a still greater price: 2.5 million children in developing countries die each year of diarrhoeal illnesses [21].

Scientists project that climate change may increase the frequency and severity of flooding [22]. Despite this and the potentially large-scale impacts caused by flooding, our understanding of the health impacts is limited, especially of longer-term effects on mental health and the health effects of lost livelihoods. The latter may prove to be even greater than the short-term impacts. For example, it has been estimated (Cairncross, unpublished data) that the total mortality directly attributable to the Mozambique floods of 2000 was less than the increase in child mortality in Mozambique expected as a result of the reduction in GDP caused by the floods. Further work is needed to develop our understanding of the health impacts, especially on mental health, and to develop better disaster preparedness and response measures.
